# Phenotype and predictors of insulin independence in adults presenting with diabetic ketoacidosis: a prospective cohort study

**DOI:** 10.1007/s00125-023-06067-3

**Published:** 2024-01-19

**Authors:** Peter J. Raubenheimer, Joanna Skelton, Bukiwe Peya, Joel A. Dave, Naomi S. Levitt

**Affiliations:** Division of Endocrinology, Department of Medicine, University of Cape Town, Cape Town, South Africa

**Keywords:** Acanthosis nigricans, Aetiology, Classification, C-peptide, Diagnosis, Insulin dependence, Insulin independence, Ketoacidosis, Ketosis-prone diabetes, Type 1 diabetes, Type 2 diabetes

## Abstract

**Aims/hypothesis:**

The aim of this work was to describe the phenotype of adults presenting with a first episode of diabetic ketoacidosis (DKA) in Cape Town, South Africa, and identify predictors of insulin independence at 12 and 60 months after presentation.

**Methods:**

A prospective, descriptive cohort study of all individuals, 18 years or older, presenting for the first time with DKA to four public-sector hospitals of the Groote Schuur Academic Health Complex was performed. Clinical, biochemical and laboratory data including GAD antibody and C-peptide status were collected at baseline. Insulin was systematically weaned and stopped in individuals who achieved normoglycaemia within the months after DKA. Individuals were followed for 12 months and then annually until 5 years after initial presentation with ketoacidosis.

**Results:**

Eighty-eight individuals newly diagnosed with diabetes when presenting with DKA were included and followed for 5 years. The mean ± SD age was 35±10 years and the median (IQR) BMI at diagnosis was 28.5 (23.3–33.4) kg/m^2^. Overall, 46% were insulin independent 12 months after diagnosis and 26% remained insulin independent 5 years after presentation. Forty-one participants (47%) tested negative for anti-GAD and anti-IA-2 antibodies and had C-peptide levels >0.3 nmol/l; in this group, 68% were insulin independent at 12 months and 37% at 5 years after diagnosis. The presence of acanthosis nigricans was strongly associated with insulin independence (OR 27.1 [95% CI 7.2, 102.2]; *p*<0.001); a positive antibody status was associated with a lower likelihood of insulin independence at 12 months (OR 0.10 [95% CI 0.03, 0.36]; *p*<0.001). On multivariable analysis only acanthosis (OR 11.5 [95% CI 2.5, 53.2]; *p*=0.004) was predictive of insulin independence 5 years after diagnosis.

**Conclusions/interpretation:**

The predominant phenotype of adults presenting with a first episode of DKA in Cape Town, South Africa, was that of ketosis-prone type 2 diabetes. These individuals presented with obesity, acanthosis nigricans, negative antibodies and normal C-peptide and could potentially be weaned off insulin at follow-up. Classic type 1 diabetes (lower weight, antibody positivity, low or unrecordable C-peptide levels and long-term insulin dependence) was less common. The simple clinical sign of acanthosis nigricans is a strong predictor of insulin independence at 12 months and 5 years after initial presentation.

**Graphical Abstract:**

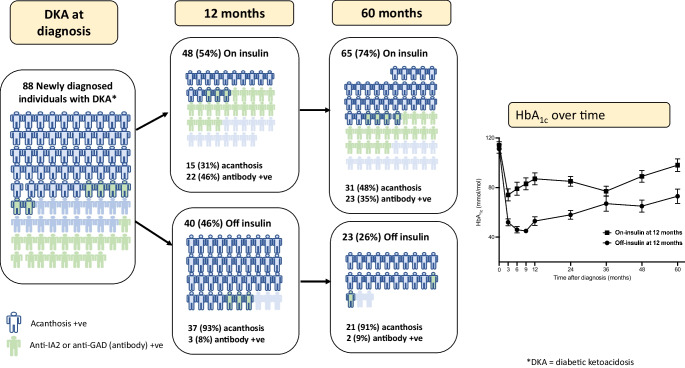

**Supplementary Information:**

The online version contains peer-reviewed but unedited supplementary material available at 10.1007/s00125-023-06067-3.


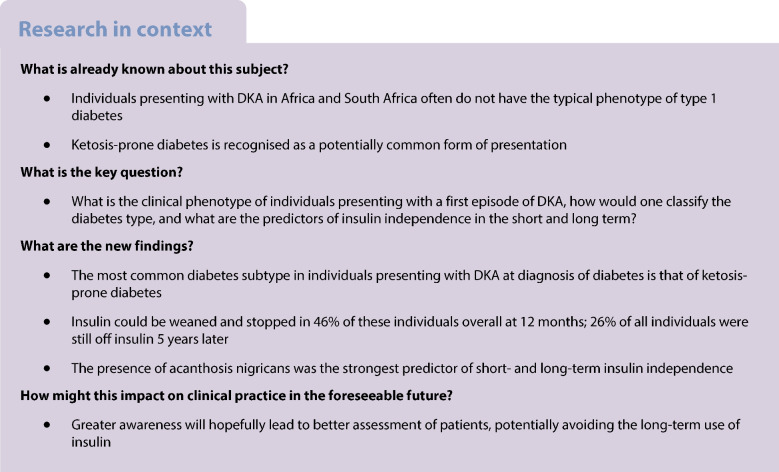


## Introduction

Diabetic ketoacidosis (DKA) is a serious hyperglycaemic emergency in individuals with diabetes and is considered a key clinical feature of type 1 diabetes. However, DKA can also develop in individuals with type 2 diabetes, under stressful conditions such as infection, post surgery or trauma [1]. In addition, DKA can be the clinical presentation of individuals newly diagnosed with type 2 diabetes without a precipitating cause [2–5]. Typical of this category of individuals, in whom the clinical presentation with severe hyperglycaemia and ketosis is similar to classic type 1 diabetes, is that they are able to stop insulin therapy and maintain glycaemic control with diet and/or oral glucose-lowering agents for some time thereafter [6–9].

There is no consensus on how to classify individuals with this form of clinical presentation, with authors variously arguing for it to be classified as a variant of type 1 diabetes or type 2 diabetes, or a sub-category called ketosis-prone type 2 diabetes (KPT2D) [2, 10–13]. The 2019 WHO Classification lists this type of presentation as a ‘hybrid diabetes’ category [14]. There is significant heterogeneity in individuals presenting with DKA and a lack of standardisation in phenotyping of participants in long-term follow-up studies. However, individuals with KPT2D generally have a low risk of recurrent ketoacidosis and the clinical course after initial DKA is much like that of individuals with type 2 diabetes rather than the diabetes being a unique subtype [12].

The clinical presentation of KPT2D was perhaps first described in case reports from central Africa in the 1950s, and subsequently in African-Americans and in France in individuals of sub-Saharan African descent [2, 11, 15]. KPT2D has since been observed in all populations, such as Chinese [5, 16], Japanese [17], Hispanic [18], Pakistani [19] and Asian-Indian [20], in children and adolescents [21, 22], being least common in populations of White ethnic background. In Africa, a few reports have identified the phenotype in Cameroon, Benin and Ivory Coast [23–25]. In South Africa, there are also individuals who present with DKA but have the phenotype of people with type 2 diabetes (e.g. high BMI [26] or normal or elevated C-peptide levels [27]). Ekpebegh et al prospectively tested 71 ‘Black African’ individuals who initially presented with DKA and classified them using the ‘Aβ’ system based on the presence of autoimmunity (A +/−) or preserved beta cell function (β +/−) [28]: 47.4% were A+β− in keeping with type 1 diabetes; however, the most common category was A−β+ (57.6%), typical of type 2 diabetes [29]. There was no follow-up of individuals to assess for predictive value of this classification system in the South African population.

Classification of individuals presenting with DKA is useful for planning future treatment strategies but this may be difficult at initial presentation, given the rising prevalence of obesity in individuals diagnosed with type 1 diabetes [30, 31] and the recognition that in certain populations KPT2D may be the most common form of diabetes in adults presenting with DKA [12]. The best performing scheme in terms of predicting a phenotype of future insulin independence is the ‘Aβ system’. This scheme has not been widely assessed and may be less reliable in other populations [32, 33]; furthermore, such a test may not be available in many low-income countries across the world.

This study aimed to describe the phenotype of individuals presenting with a first episode of DKA and the prospective long-term follow-up of such individuals to further compliment the admission phenotype, and to describe predictors of insulin independence in these individuals.

## Methods

### Study design and population

This was a prospective, descriptive survey of all individuals, aged 18 years or older, presenting for the first time with DKA to four hospitals of the Groote Schuur Hospital Academic Health Complex in Cape Town, South Africa. Recruitment took place from mid-2013 to mid-2016, with the exception of a 6 month period in 2015 when research staff were not available. Both newly diagnosed and previously diagnosed individuals with diabetes were initially included. DKA was defined by the presence of all of the following: serum glucose >13.9 mmol/l, pH <7.3, serum bicarbonate concentration <18 mmol/l and documented ketonaemia or ketonuria (measured with semi-quantitative assay of acetoacetate), together with the absence of concomitant conditions that might result in similar ketoacidosis such as pregnancy, acute alcohol intoxication and organic poison ingestion. Individuals were acutely managed at the hospital of presentation with standard protocols of intravenous fluids and insulin, and were referred to the Groote Schuur Hospital diabetes clinic 3 weeks after discharge, where the study team assumed all subsequent outpatient care.

The study was approved by the University of Cape Town Faculty of Health Sciences Human Research Ethics Committee (HREC REF 417/2011). All participants gave informed consent.

### Data collection and follow-up

Those who met entry criteria underwent a detailed assessment of medical history, precipitating causes of DKA, clinical examination and serum and urine biochemistry. Precipitating causes were sought for history, examination findings, blood tests, blood and urine culture and routine chest x-rays.

Ethnic background was self-reported by the participants, and ethnicity (Black African, Coloured [a mixed ethnic background], White and Indian/Asian) was assigned by self-classification into one of the four formally recognised South African population groups as used by the Department of Statistics of South Africa for data reporting [34]. A standard outpatient diabetes management protocol was followed. Participants were placed on twice-daily regular pre-mixed insulin (Actraphane; Novo Nordisk, Denmark) and metformin was added for those with a BMI >25 kg/m^2^ at the first outpatient visit. Participants were instructed in measuring capillary blood glucose levels at least twice daily. Subsequent follow-up was every 2–4 weeks initially or as required (both in the clinic or by telephone), with regular study reviews at 1 month, and then every 3 months for the first year. If mean capillary glucose levels during a two-week period were at target (fasting glucose <7 mmol/l and postprandial glucose <10 mmol/l), the insulin was reduced by 50%; if capillary glucose remained at target during the next 2 weeks, the insulin was discontinued and metformin introduced or, if already prescribed, continued at the same dose. Participants were followed for 1 year after the initial visit, with subsequent treatment strategies following usual clinical guidance, with addition of either a sulfonylurea or reintroduction of insulin as needed based on HbA_1c_ measurements. After 1 year of study follow-up, participants were returned to their usual care (either in primary care or at hospital diabetes clinics). Participants were followed up thereafter annually by clinic visit, telephone or review of electronic records, electronic prescriptions and laboratory tests.

HbA_1c_ was measured by turbidimetric inhibition immunoassay (Roche Diagnostics, Rotkreuz, Switzerland)). Serum was analysed for the presence of GAD-65 antibodies and human antibodies against tyrosine phosphatase (IA2) in serum using the EUROIMMUN ELISA IgG kits (EUROIMMUN Medizinische Labordiagnostika, Lübeck, Germany), and values of >10 U/ml were considered positive for either. Beta cell secretory capacity was assessed by measuring C-peptide in the overnight fasting state, at least 12 h after the last injection of insulin, using the Siemens Immulite 2000 system (Siemens Healthcare, Erlangen, Germany). An Aβ classification of ‘beta cell positive’ was given if fasting C-peptide was >0.3 nmol/l.

### Statistical analysis

Data management and statistical analyses were conducted in SPSS version 28.0 (IBM SPSS Statistics, Armonk, NY, USA). Graphs were drawn in Prism (GraphPad Software, Boston, MA, USA). The characteristics of participants according to baseline variables obtained at admission or at first study visit were summarised and compared using descriptive statistics with *p*<0.05 considered significant; categorical variables are reported as count and percentage while continuous variables were first tested by Shapiro–Wilk test for normality and are reported as means and SD or median and IQR as appropriate. Characteristics of participants who were ‘off insulin’ 12 months after the initial episode of DKA were compared with those who remained on insulin. Predictive factors for insulin discontinuation were described using a univariate logistic regression analysis calculating the ORs and 95% CIs for being off insulin. For multivariable analysis, variables that were significant at the *p*<0.157 level [35] and/or those that were deemed clinically significant based on our experience and that of previous publications were all included in an initial global model, after which all significant variables were included in a second model. Various methods to describe residual insulin secretion using the fasting C-peptide level were used: using C-peptide as a continuous variable; using cut-points of 0.2 nmol/l or 0.3 nmol/l; or using a fasting C-peptide/glucose ratio. The multivariable model used cut-points commonly used in clinical practice (0.2 and 0.3 nmol/l). Characteristics between Aβ groups were performed by *χ*^2^ test for categorical variables and Kruskal–Wallis test for continuous variables with a Dunn post hoc test for pairwise comparisons.

## Results

Of the 118 individuals who were admitted with a first episode of DKA and referred to the clinic for follow-up, 88 are included in this report. Participants were excluded for the following reasons: one person died (suspected malignancy); four became pregnant within the first 12 months after diagnosis; six moved to another province or country; and four were lost to follow-up. Five participants with previously diagnosed type 2 diabetes were already on insulin before admission and, with all having diabetes of prolonged duration and HbA_1c_ >86 mmol/mol (10%), no attempt was made to wean them off insulin; these individuals are not included in our results. Only ten participants were previously diagnosed with type 2 diabetes and not on insulin at the time of their DKA admission, so we excluded them from further analysis, and only focused on the 88 participants with newly diagnosed diabetes. Electronic supplementary material (ESM) Table 1 contrasts the new vs previously diagnosed people with diabetes.

### Baseline results

The mean + SD age was 35 ± 10 years (with a maximum age of 61 years), 57% were male and their self-identified racial identity was 57% Black and 43% Coloured. At presentation, the mean HbA_1c_ was 113 mmol/mol (12.5%); 16 (18%) presented with severe DKA (pH <7.0), 54 (61%) with moderate DKA (pH 7.0–7.24) and 18 (21%) with mild DKA (pH 7.25–7.29).

### Comparison of groups 12 months after diagnosis

Overall, 40 participants (46%) did not require insulin therapy 12 months after initial admission. Table 1 describes the baseline clinical, phenotypic and laboratory characteristics of the on-insulin and off-insulin groups. The mean age was significantly higher in the off-insulin group (38±10 years) compared with 33±10 years in the on-insulin group; *p*=0.014. There were no differences in the on- or off-insulin groups regarding sex, self-identified ethnicity, clinical history in terms of potential risk factors, associations for developing diabetes, for precipitants of DKA, admission blood glucose or HbA_1c_. At admission with DKA, the median (IQR) blood glucose was 27.9 (23.6–44.0) mmol/l and the mean ± SD HbA_1c_ was 112±21 mmol/mol (12.4±1.9%). However, the off-insulin group had less severe acidosis, measured by pH or serum bicarbonate, and had a higher BMI (median [IQR] 32.0 [28.3–36.2] kg/m^2^ vs 25.1 [22.0–31.0]; *p*<0.001); only five participants in the off-insulin group had a BMI <24.9 kg/m^2^ vs 22 participants in the on-insulin group (*p*<0.001). Acanthosis nigricans was present in 93% of the off-insulin group compared with 31% in the on-insulin group (*p*<0.001). At follow-up 2–3 weeks after admission, the off-insulin group had a higher fasting C-peptide level, and C-peptide/glucose ratio (*p*<0.001). Only one participant in the off-insulin group was GAD antibody positive vs 19 (40%) in the on-insulin group (*p*<0.001). Anti-IA-2 was not discriminatory.

**Table 1:** Baseline characteristics of participants admitted to the clinic with DKA, classified into those on or off insulin 12 months after presentation

Characteristic	All (*n*=88)	On insulin (*n*=48)	Off insulin (*n*=40)	*p* value
Demographics				
Age, years	35±10	33±10	38±10	0.014
Male sex	50 (57)	25 (52)	25 (63)	0.326
Ethnicity				
Black	50 (57)	25 (52)	25 (63)	0.390
Mixed ethnicity (Coloured)^a^	38 (43)	23 (48)	15 (38)	
Clinical history				
Family history of diabetes	46 (52)	23 (48)	23 (58)	0.370
History of other metabolic disease^b^	16 (18)	7 (15)	9 (23)	0.338
HIV diagnosis	4 (5)	1 (2)	3 (8)	0.326
History of smoking^c^	33 (38)	17 (35)	16 (40)	0.658
History of alcohol use^c^	42 (48)	25 (52)	17 (43)	0.399
Precipitant identified	22 (25)	13 (27)	9 (23)	0.621
Precipitated by infection	20 (23)	12 (25)	8 (20)	0.740
Examination				
BMI, kg/m^2^	28.5 (23.3–33.4)	25.1 (22.0–31.0)	32.0 (28.3–36.2)	<0.001
BMI <18.5 kg/m^2^	3 (3)	3 (5)	0	0.193
BMI 18.5–24.9 kg/m^2^	24 (27)	19 (40)	5 (13)	0.004
BMI 25–29.9 kg/m^2^	22 (25)	13 (27)	9 (23)	0.404
BMI 30–39.9 kg/m^2^	27 (31)	9 (19)	18 (45)	0.007
BMI >40 kg/m^2^	12 (14)	4 (8)	8 (20)	0.101
Acanthosis nigricans present	52 (59)	15 (31)	37 (93)	<0.001
Systolic BP (mmHg)	127±17	125±18	131±14	0.075
Diastolic BP (mmHg)	79 ±12	78 ±10	84 ±12	0.006
Admission investigations				
Glucose, mmol/l	27.9 (23.6–44.0)	28.7 (23.3–44.0)	27.8 (24.0–37.8)	0.900
pH	7.15 (7.04–7.22)	7.10 (7.00–7.18)	7.20 (7.11–7.25)	<0.001
Bicarbonate, mmol/l	10.0 (6.8–12.3)	8.4 (6.4–11.1)	12.2 (7.5–14.5)	0.001
Investigations				
HbA_1c_, mmol/mol	112±21	115±21	111±22	0.386
HbA_1c_, %	12.4±1.9	12.7±1.9	12.3±2.0	0.386
Creatinine, μmol/l	62±15	60±16	64±15	0.041
Total cholesterol, mmol/l	4.9±1.2	5.0±1.1	5.0±1.3	0.983
Triglycerides, mmol/l	1.6±0.7	1.5±0.8	1.8±0.7	0.010
HDL-cholesterol, mmol/l	1.3±0.5	1.5±0.6	1.2±0.4	0.024
LDL-cholesterol, mmol/l	2.9±1.0	2.9±0.9	3.0±1.1	0.440
Triglyceride/HDL-cholesterol ratio	1.4±1.0	1.3±1.2	1.7±0.9	0.004
Bloods phenotype				
Fasting plasma glucose, mmol/l	11.2±6.6	11.5±9.6	7.8±5.4	0.007
Fasting C-peptide, nmol/l	0.33 (0.17–0.62)	0.23 (0.13–0.23)	0.45 (0.30–0.70)	<0.001
C-peptide/glucose, nmol/mmol×100	3.43 (1.47–6.83)	2.34 (0.98–4.32)	5.95 (3.10–10.48)	<0.001
Fasting C-peptide >0.3 nmol/l	50 (57)	19 (40)	31 (78)	<0.001
Fasting C-peptide >0.2 nmol/l	65 (74)	29 (60)	36 (90)	<0.001
Anti-GAD positive (>10 U/ml)	20 (23)	19 (40)	1 (3)	<0.001
Anti-IA-2 positive (>10 U/ml)	11 (13)	9 (19)	2 (5)	0.061
Antibody (GAD or IA-2) positive	25 (28)	22 (46)	3 (8)	<0.001

Data are mean ± SD, *n* (%) or median (IQR) unless otherwise stated

^a^Ethnicity was self-identified based on current official South African population group classification

^b^History of hypertension, peripheral vascular disease, cerebrovascular disease, ischaemic heart disease, dyslipidaemia, polycystic ovary syndrome or gestational diabetes

^c^Any use at all in the last year

*p* value is for Mann–Whitney *U* test for continuous variables and *χ*^2^ test or Fisher’s exact for the categorical variables

Univariate predictors of the baseline data for being off insulin 12 months after presentation with DKA are shown in Table 2. Statistically significant predictors included older age, a higher BMI, the presence of acanthosis nigricans, higher diastolic BP, less severe acidosis on admission, higher triglycerides, a lower HDL-cholesterol, a lower glucose and higher fasting C-peptide at 3 weeks after admission and the absence of anti-GAD and anti-IA-2 antibodies. The strength of these associations was tested in the multivariate model, with only the presence of acanthosis nigricans and the absence of antibodies (anti-GAD or anti-IA-2) remaining significant predictors of being off insulin at 12 months after diagnosis (Table 3).

**Table 2:** Univariate predictors for individuals on/off insulin at 12 months and at 60 months after presenting with DKA

Characteristic	At 12 months				At 60 months			
	On insulin (*n*=48)	Off insulin (*n*=40)	OR (95% CI)	*p* value	On insulin (*n*=65)	Off insulin (*n*=23)	OR (95% CI)	*p* value
Demographics								
Age, years	33±10	38±10	1.06 (1.01, 1.10)	0.019	35±11	36±9	1.01 (0.97, 1.06)	0.554
Male sex	25 (52)	25 (63)	1.53 (0.65, 3.60)	0.327	36 (55)	14 (61)	1.25 (0.48, 3.31)	0.648
Ethnicity								
Black	25 (52)	25 (63)	0.65 (0.28, 1.53)	0.327	33 (51)	17 (74)	2.75 (0.96, 7.85)	0.059
Mixed ethnicity (Coloured)^a^	23 (48)	15 (38)	1.53 (0.65, 3.60)	0.327	32 (49)	6 (26)	0.36 (0.13, 1.04)	0.059
Clinical history								
Family history of diabetes	23 (48)	23 (58)	1.47 (0.63, 3.43)	0.370	33 (51)	13 (57)	0.79 (0.31, 2.07)	0.635
History of other metabolic disease^b^	7 (15)	9 (23)	1.70 (0.57, 5.07)	0.338	11 (17)	5 (22)	0.73 (0.22, 2.40)	0.608
HIV diagnosis	1 (2)	3 (8)	0.26 (0.03, 2.63)	0.255	3 (5)	1 (4)	1.07 (0.11, 10.78)	0.958
History of smoking^c^	17 (35)	16 (40)	1.22 (0.51, 2.89)	0.658	29 (45)	4 (17)	0.26 (0.08, 0.85)	0.026
History of alcohol use^c^	25 (52)	17 (43)	0.68 (0.29, 1.58)	0.370	35 (54)	7 (30)	2.67 (0.97, 7.35)	0.058
Precipitant identified	13 (27)	9 (23)	0.78 (0.29, 2.08)	0.244	17 (26)	5 (22)	1.28 (0.41, 3.97)	0.675
Examination								
BMI, kg/m^2^	25.1 (22.0–31.0)	32.0 (28.3–36.2)	1.12 (1.04, 1.20)	0.002	27.3 (22.6–32.9)	32.0 (28.4–35.0)	1.04 (0.98, 1.09)	0.178
BMI <18.5 kg/m^2^	3 (5)	0	NA	NA	3 (5)	0	NA	NA
BMI 18.5–24.9 kg/m^2^	19 (40)	5 (13)	0.22 (0.07, 0.67)	0.005	22 (34)	2 (9)	0.19 (0.04, 0.87)	0.032
BMI 25–29.9 kg/m^2^	13 (27)	9 (23)	0.78 (0.29, 2.07)	0.621	17 (26)	5 (22)	0.78 (0.26, 2.44)	0.675
BMI 30–39.9 kg/m^2^	9 (19)	18 (45)	3.55 (1.36, 9.22)	0.009	14 (22)	13 (57)	4.73 (1.72, 13.06)	0.003
BMI >40 kg/m^2^	4 (8)	8 (20)	2.75 (0.76, 9.93)	0.122	9 (14)	3 (13)	0.93 (−0.23, 3.80)	0.923
Acanthosis nigricans present	15 (31)	37 (93)	27.13 (7.21, 102.13)	<0.001	31 (48)	21 (91)	11.52 (2.50, 53.18)	0.002
Systolic BP, mmHg	125±18	131±14	1.02 (0.99, 1.05)	0.105	126±17	131±15	1.02 (0.99, 1.05 )	0.24
Diastolic BP, mmHg	78±10	84±12	1.07 (1.03, 1.12)	0.003	78±10	84±14	1.05 (1.00, 1.10)	0.035
Admission investigations								
Glucose, mmol/l	28.7 (23.3–44.0)	27.8 (24.0–37.8)	0.99 (0.96, 1.03)	0.585	27.8 (24.0–44.0)	29.0 (24.0-44.0)	1.01 (0.97, 1.05)	0.689
pH	7.10 (7.00-7.18)	7.20 (7.11-7.25)	262.38 (6.68, 10,225.56)	0.003	7.11 (7.02-7.19)	7.22 (7.15–7.27)	186.50 (2.21, 15,769.73)	0.021
Bicarbonate, mmol/l	8.4 (6.4–11.1)	12.2 (7.5–14.5)	1.17 (1.05, 1.31)	0.006	8.8 (6.5–11.5)	12.3 (10.2–15.0)	1.24 (1.07, 1.43)	0.004
Investigations								
HbA_1c_, mmol/mol	115±21	111±22			113±21	110±23		
HbA_1c_, %	12.7±1.9	12.3±2.0	0.90 (0.72, 1.13)	0.368	12.5±1.9	12.2±2.1	0.93 (0.73, 1.19)	0.571
Creatinine, μmol/l	60±16	64±15	1.02 (0.99, 1.05)	0.131	62±15	63±17	1.01 (0.98, 1.04)	0.649
Total cholesterol, mmol/l	5.0±1.1	5.0±1.3	1.03 (0.71, 1.49)	0.865	5.0±1.1	4.6±1.1	0.70 (0.45, 1.09)	0.117
Triglycerides, mmol/l	1.5±0.8	1.8±0.7	2.04 (1.09, 3.83)	0.026	1.6±1.1	1.5±0.6	0.97 (0.51, 1.85)	0.924
HDL-cholesterol, mmol/l	1.5±0.6	1.2±0.4	0.31 (0.12, 0.84)	0.020	1.4±0.6	1.2±0.5	0.47 (0.16, 1.38)	0.171
LDL-cholesterol, mmol/l	2.9±0.9	3.0±1.1	1.18 (0.78, 1.78)	0.446	2.9±1.0	2.7±1.1	0.78 (0.48, 1.27)	0.320
Triglyceride/HDL-cholesterol ratio	1.3±1.2	1.7±0.9	1.41 (0.92, 2.16)	0.113	1.4±1.1	1.5±0.9	1.06 (0.69, 1.65)	0.785
Bloods phenotype								
Fasting plasma glucose, mmol/l	11.5±9.6	7.8±5.4	0.88 (0.98, 0.96)	0.005	11.5±1.1	8.4±4.1	0.88 (0.79, 0.99)	0.028
Fasting C-peptide, nmol/l	0.23 (0.13–0.23)	0.45 (0.30–0.70)	2.56 (1.44, 4.55)	0.001	0.30 (0.17–0.51)	0.43 (0.26–0.69)	1.66 (1.06, 2.58)	0.026
C-peptide/glucose, nmol/mmol × 100	2.34 (0.98–4.32)	5.95 (3.10–10.48)	1.29 (1.11, 1.48)	<0.001	3.10 (1.43–5.40)	5.07 (2.63–10.54)	1.13 (1.02, 1.24)	0.017
Fasting C-peptide >0.3 nmol/l	19 (40)	31 (78)	5.26 (2.05, 13.47)	<0.001	33 (51)	17 (74)	2.75 (0.91, 7.85)	0.059
Fasting C-peptide <0.2 nmol/l	19 (40)	4 (10)	0.17 (0.05, 0.55)	0.003	19 (29)	4 (17)	0.51 (0.15, 1.70)	0.272
Anti-GAD positive (>10 U/ml)	19 (40)	1 (3)	0.04 (0.01, 0.31)	<0.001	19 (29)	1 (4)	0.11 (0.01, 0.88)	0.037
Anti-IA-2 positive (>10 U/ml)	9 (19)	2 (5)	0.23 (0.05, 1.13)	0.052	10 (15)	1 (4)	0.25 (0.03, 2.07)	0.199
Antibody (GAD or IA-2) positive	22 (46)	3 (8)	0.10 (0.03, 0.36)	<0.001	23 (35)	2 (9)	0.17 (0.04, 0.81)	0.026

Data are mean ± SD, *n* (%) or median (IQR) unless otherwise stated

^a^Ethnicity was self-identified based on current official South African population group classification

^b^History of hypertension, peripheral vascular disease, cerebrovascular disease, ischaemic heart disease, dyslipidaemia, polycystic ovary syndrome or gestational diabetes

^c^Any use at all in the year before admission

*p* value is for Mann–Whitney *U* test for continuous variables and *χ*^2^ test or Fisher's exact for the categorical variables

**Table 3:** Predictors from the multivariable model of being off insulin 12 months after diagnosis with DKA

Variable	Step 1 (global model)		Step 2	
	OR (95% CI)	*p* value	OR (95% CI)	*p* value
Demographics				
Age	1.10 (1.00, 1.20)	0.046		
Precipitant identified	2.21 (0.47, 9.54)	0.329		
Examination				
BMI	0.98 (0.90, 1.07)	0.634		
Acanthosis nigricans present	69.42 (5.47, 881.36)	0.001	26.24 (5.58, 123.48)	<0.001
Diastolic BP	1.04 (0.96, 1.13)	0.312		
Admission Investigations				
Serum standard bicarbonate, mmol/l	1.08 (0.92, 1.28)	0.333		
Investigations				
Triglycerides	1.64 (0.53, 5.08)	0.393		
HDL-cholesterol	3.56 (0.52, 24.43)	0.197		
Bloods phenotype				
Fasting C-peptide >0.3 nmol/l	3.78 (0.53, 26.89)	0.184		
Fasting C-peptide <0.2 nmol/l	0.14 (0.01, 2.20)	0.162		
Anti-GAD or anti-IA-2 positive (>10 U/ml)	0.09 (0.01, 0.66)	0.017	0.15 (0.03, 0.83)	0.030

*χ*^2^ (2)=49.006, *p*<0.001; *R*^2^= 0.571

### Comparison of groups after 5 years of follow-up

Five years after initial presentation with DKA, 23 participants (26% of the initial cohort) remained off insulin and none presented with recurrent DKA. Univariate predictors from baseline data of stopping and remaining off insulin for 5 years are shown in Table 2 and multivariable predictors are shown in Table 4. On univariate analysis, participants who remained off insulin at 5 years presented with less severe acidosis (measured by pH or bicarbonate) during the initial DKA admission, were more likely to be obese (BMI 30–39.9 kg/m^2^) and acanthosis nigricans was present more often; fasting C-peptide was higher and anti-GAD antibodies were negative (in all except one participant). On multivariate analysis, however, only the presence of acanthosis nigricans remained predictive of remaining off insulin.

**Table 4:** Multivariate predictors for remaining off insulin 5 years after diagnosis with DKA in newly diagnosed diabetes mellitus

Variable	Step 1 (global model)		Step 2	
	OR (95% CI)	*p* value	OR (95% CI)	*p* value
Demographics				
Age	0.99 (0.93, 1.07)	0.916		
Black (vs Coloured)	0.69 (0.14, 3.31)	0.646		
History of smoking	0.26 (0.05, 1.42)	0.120		
History of alcohol	0.32 (0.7, 1.4)	0.133		
Examination				
BMI	0.91 (0.79, 1.04)	0.171		
Acanthosis nigricans present	67.13 (3.72, 1211.50)	0.004	11.52 (2.49, 53.18)	0.002
Diastolic BP	1.04 (0.97, 1.11)	0.306		
Admission investigations				
Bicarbonate	1.18 (0.97, 1.40)	0.062		
Bloods phenotype				
Total cholesterol	0.81 (0.47, 1.40)	0.488		
Fasting C-peptide >0.3 nmol/l	2.00 (0.24, 16.96)	0.522		
Fasting C-peptide < 0.2 nmol/l	0.56 (0.08, 3.99)	0.104		
Anti-GAD or anti-IA-2 positive (>10 U/ml)	0.55 (0.08, 3.86)	0.565		

*χ*^2^ (1)=13.37, *p*<0.001; *R*^2^= 0.237

### Comparison using the Aβ classification system

ESM Table 2 presents the data classified into the ‘Aβ’ classification system. There were 41 (47%) participants in the antibody-negative C-peptide positive (A−β+) group and 16 (18%) in the antibody-positive, C-peptide-negative (A+β−) group. The four groups were phenotypically significantly different from each other with respect to BMI, the presence of acanthosis nigricans, severity of acidosis at initial presentation with DKA and lipid profile (HDL-cholesterol and triglycerides). At 12 months after diagnosis, 28 (68%) participants with the A−β+ phenotype were off insulin compared with none of the participants in the A+β− group, nine (41%) in the A−β− group and three (33%) in the A+β− group. Thirty-seven per cent of the A−β+ group remained off insulin at 5 years of follow-up.

**Fig. 1:**
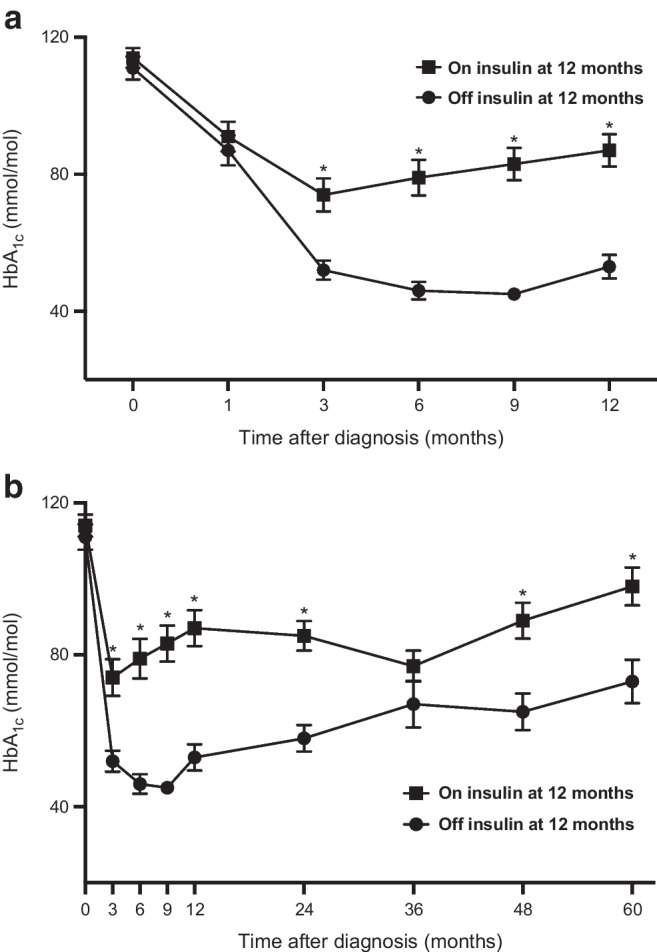
HbA_1c_ over time for the off-insulin group (*n*=40) vs the on-insulin group (*n*=48) at 12 months (**a**) and at 60 months (**b**) after initial presentation with DKA. Data are shown as mean ± SEM. *p<0.001

Over the 12 months of follow-up, the HbA_1c_ of participants in whom insulin could be stopped (the off-insulin group) vs those in whom insulin could not be stopped (the on-insulin group) diverged at 3 months and remained significantly different, with a mean ± SD HbA_1c_ at 3 months in the off-insulin group of 50±10 mmol/mol (6.7±0.9%) vs 78±34 mmol/mol (9.3±3.1%) in the on-insulin group (*p*<0.001) (Fig. 1). Differences were less apparent between the Aβ groups; only the A−β+ group was different from the A+β− group at 3, 6 and 9 months after diagnosis but there was no difference by 12 months (ESM Fig. 1). Adding the 3 or 6 month HbA_1c_ level to the multivariable models to predict insulin independence at 12 or 60 months did not contribute or alter the models significantly.

## Discussion

In this cohort of adults with newly diagnosed diabetes presenting with DKA, the commonest phenotype was that of type 2 diabetes, or the A−β+ phenotype in the Aβ classification [28]. The majority were overweight and had no obvious precipitant for DKA. Overall, 46% of the participants did not require insulin 12 months after diagnosis and 26% still did not require insulin 5 years after diagnosis; in the A−β+ group 68% remained off insulin at 12 months and 37% at 5 years. Predictors of not requiring insulin after 12 months included older age, presence of acanthosis nigricans and the absence of anti-GAD antibodies. Only the presence of acanthosis nigricans remained a useful predictor for not requiring insulin 5 years after diagnosis. After discontinuation of insulin, there was, however, progressive decline in glycaemic control over time as might be expected in people with type 2 diabetes.

This is the first prospective study from sub-Saharan Africa describing the phenotype of adults newly diagnosed with diabetes who present with DKA and is one of few studies worldwide where insulin withdrawal was systematically attempted. It is one of only three studies worldwide with follow-up of a substantial number of individuals beyond 12 months after diagnosis. Since the first prospective follow-up description by Umpierrez in 1995 [11], in which it was reported that 25/46 (54%) of all individuals presenting with DKA to Grady Memorial Hospital in Atlanta were able to discontinue insulin and were insulin independent 12 months post admission, seven other studies have reported on insulin independence 12 months after presentation with ketosis or ketoacidosis. Entry criteria were not directly comparable and recruitment was sometimes targeted to individuals predicted to have KPT2D but overall between 27% and 72% of individuals presenting with DKA (or ketosis only in some studies) were insulin independent at 12 months. Umpierrez et al [36] reported on 17 patients with obesity and with DKA, in whom insulin was stopped but overall insulin independence rates at 12 months were not specified although the report comments on prolongation of normoglycaemia after use of a sulfonylurea. Maldonado et al [3] reported on 103 individuals presenting with DKA (41 newly diagnosed with diabetes) recruited from Ben Taub hospital in Houston, Texas with an ethnic distribution of 40% Hispanic-American, 44% African-American, 15% White-American and 1% Asian-American. At 12 months after diagnosis, 31/103 (30%) of all individuals with DKA were off insulin. Beta cell reserve at the time of DKA was the strongest predictor of being able to wean and stop insulin and of future glycaemic control. In France in 2004, Mauvais-Jarvis et al [2] reported on 132 individuals of sub-Saharan African origin newly diagnosed with ketosis or DKA (68 had DKA but were not separately analysed). Insulin could be weaned and stopped in 64% of the individuals at a mean of 14.3 weeks after initial presentation. After 10 years of follow-up, 34 of the cohort of 84 (40%) in whom insulin was stopped were still off insulin. This study included individuals with ketosis and not exclusively ketoacidosis, so the data are not directly comparable with the present study. In a second report from that institution by Balasubramanyam [28], with a larger study group of 294 individuals (135 with newly diagnosed diabetes), 31% of the cohort were successfully withdrawn from insulin therapy and remained off insulin at 12 months. ROC analysis revealed that the Aβ classification system best predicted whether insulin could be discontinued. Seok et al in Korea (2013) [37] followed 44 individuals presenting with DKA for at least 12 months, at which stage 12 (27%) were off insulin. In this study, 4 year follow-up data for 16 individuals were reported, with four still off insulin. Vellanki et al (2016) [38], in a trial of sitagliptin vs metformin and placebo in pre-selected African-American individuals with overweight or obesity in Atlanta USA with new or hyperglycaemia and unprovoked ketosis or DKA and who had insulin discontinued within 12 weeks of initial presentation, reported a 2 year overall insulin independence rate of 58%. Gupta (2017) [20] reported on 51 individuals with newly diagnosed diabetes admitted with DKA to the Christian Medical College in Vellore, India. Thirteen individuals were considered to have KPT2D based on negative antibodies and the absence of an obvious precipitant. In 11 of those insulin was stopped and they remained off insulin at 12 months (2 were lost to follow-up). Overall, of the 51 admitted individuals, 21% were off insulin at 12 months.

The clinical entity of KPT2D is characterised by DKA at first presentation with diabetes in people with obesity, usually of middle age, with no obvious precipitating cause and the absence of anti-GAD and anti-IA-2 antibodies. Although the presence of acanthosis nigricans in the neck has previously been described in case reports, it has not been included as a finding in previously published large series of individuals with KPT2D. Not only is it a very useful sign of the diabetes subtype of KPT2D but it is also the best predictor of short- and long-term independence from insulin. Others have suggested that skin tags may be a useful feature of this syndrome, although it is not clear that this finding will offer any additional prediction beyond acanthosis nigricans, which was present in all individuals with skin tags [39]. We did not specifically assess for the presence of skin tags in this study.

Predicting which individuals could be weaned off insulin is helpful in settings where resources may not allow regular careful follow-up (as offered to participants in this study) and careful selection for follow-up and weaning may be helpful. In addition, measurement of anti-GAD and anti-IA-2 antibodies, and of C-peptide, may not be available in many parts of Africa and the world making a clinical sign that is predictive of KPT2D useful.

This study has several limitations and confirmation in other populations is important. The population was recruited from the Cape Town City Metropole only, and these findings may not apply to rural areas or to other areas of Africa, with its large genetic and phenotypic variability of type 2 diabetes [40, 41]. In addition, this study did not recruit any White individuals, so this study was unable to ascertain whether ethnicity is a strong predictor of insulin independence. We did not have access to the zinc transporter 8 antibody assay, which may have improved identification of a few more people with type 1 diabetes. However, the positivity rate in South Africa is likely to be much lower than in a European population [42].

In conclusion, the predominant phenotype of adults presenting with newly diagnosed diabetes and DKA in Cape Town, South Africa, is that of KPT2D. Thus, many adults with diabetes diagnosed at the time of presenting with DKA (‘ketosis-onset diabetes’), especially those with an obese phenotype with acanthosis nigricans, and absence of anti-GAD antibodies, could potentially be weaned off insulin safely using a standardised protocol. Almost one-third of such individuals could continue to be managed without insulin for 5 years, thus avoiding the extra burden, potential risks and cost of insulin therapy, at least for some time. Consideration should be given to replicate this work in other countries in Africa and to develop region-specific guidelines on how to recognise and implement insulin weaning strategies. In future, phenotypic and genotypic classification systems may enable better aetiological diagnosis of diabetes and better strategies toward ideal, individualised treatment.

### Supplementary Information

Below is the link to the electronic supplementary material.

Supplementary file1 (PDF 111 KB)

## Data Availability

The data that support the findings of this study are not openly available due to reasons of sensitivity and are available from the corresponding author upon reasonable request.

## Abbreviations

DKA: Diabetic ketoacidosis
KPT2D: Ketosis-prone type 2 diabetes
