# Supplementary material for: Phenotype and predictors of insulin independence in adults presenting with diabetic ketoacidosis: a prospective cohort study
Source: Diabetologia. 2024 Jan 19;67(3):494–505. doi: 10.1007/s00125-023-06067-3 (PMC10844464; doi:10.1007/s00125-023-06067-3)
Supplement: Supplementary file 1 — Supplementary file1 (PDF 111 KB) [file 125_2023_6067_MOESM1_ESM.pdf]

Electronic Supplementary Material

ESM Table 1. New onset vs previously-diagnosed diabetes: baseline demographics

Characteristic	All	New-onset DM	Previously diagnosed DM	P
	n=103	n=88	n=15	
**Demographics**				
Age (years)	35±16	34±14	47±13	< 0.001
Male sex	55 (53)	50 (57)	5 (33)	0.08
Ethnicity				0.577
Black	60 (58)	50 (57)	10 (67)	
Mixed ethnicity (SA coloured)[a]	43 (42)	38 (43)	5 (33)	
**Clinical history**				
Family history of diabetes	52 (51)	46 (52)	6 (40)	0.416
History of other metabolic disease[b]	22 (21)	16 (6)	6 (405)	0.007
HIV diagnosis	5 (5)	4 (5)	1 (7)	0.552
History of smoking[†]	35 (34)	33 (38)	2 (13)	0.082
History of alcohol use[†]	47 (46)	42 (48)	5 (33)	0.403
Precipitant identified	32 (31)	22 (25)	10 (67)	0.002
Precipitated by infection	28 (27)	20 (23)	8 (53)	0.012
**Examination**				
BMI (kg/m^2)	28.3 (23.0-33.2)	28.5 (23.3-33.4)	25.8 (22.3-31.9)	0.253
BMI < 18.5 kg/m^2	3 (3)	3 (3)	0	0.62
BMI 18.5 - 24.9 kg/m^2	30 (30)	24 (28)	6 (43)	0.239
BMI 25 - 29.9 kg/m^2	26 (25)	22 (25)	4 (27)	0.558
BMI 30 - 39.9 kg/m^2	30 (29)	27 (31)	3 (20)	0.305
BMI > 40 kg/m^2	13 (13)	12 (14)	1 (7)	0.4
Acanthosis nigricans present	58 (56)	52 (59)	6 (40)	0.26

Systolic blood pressure (mmHg)	126±17	127±17	119±16	0.093
Diastolic blood pressure (mmHg)	79±11	79±12	76±11	0.203
**Admission Investigations**				
Glucose (mmol/l)	27.8 (23.2-44.0)	27.9 (23.6-44.0)	26.0 (19.4-44.0)	0.385
pH	7.15 (7.04-7.22)	7.15 (7.04-7.22)	7.15 (7.01-7.23)	0.866
Bicarb (mmol/l)	9.8 (6.7-12.3)	27.9 (23.6-44.0)	9.8 (5.8-11.7)	0.716
**Investigations**				
HbA_{1c} (mmol/mol)	113±22	113±21	123±22	
HbA_{1c} (%)	12.5±2.0	12.4±1.9	13.4±2.0	0.059
Creatinine (umol/l)	61.9±15.7	62.0±15.3	61.1±18.4	0.457
Total cholesterol (mmol/l)	5.0±1.2	4.9±1.2	5.5±1.2	0.088
Triglycerides (mmol/l)	1.6±0.8	1.6±0.7	1.7±1.0	0.712
HDL cholesterol (mmol/l	1.4±0.5	1.3±0.5	1.5±0.5	0.237
LDL cholesterol (mmol/l)	2.9±1.0	2.9±1.0	3.3±0.7	0.092
Trig/HDL Ratio	1.2±1.0	1.2±1.2	1.1±0.4	0.783
**Bloods phenotype**				
Fasting plasma glucose (mmol/l)	9.7±9.1	11.2 ± 6.6	12.9±7.9	0.189
Fasting c-peptide (nmol/L)	0.30 (0.17-0.53)	0.33 (0.17-0.62)	0.20 (0.07-0.50)	0.074
C-peptide:glucose (nmol/mmol x 100)	3.13 (1.45-6.00)	3.43 (1.47-6.83)	2.02 (0.89-3.84)	0.015
Fasting c-peptide > 0.3 nmol/l	54 (53)	50 (57)	4 (29)	0.081
Fasting c-peptide > 0.2 nmol/l	74 (72)	65 (74)	9 (60)	0.351
Anti-GAD +ve (>10 IU/ml)	23 (22)	20 (23)	3 (20)	0.558
Anti-IA2 +ve (> 10 IU/ml)	11 (11)	11 (13)	0	0.36
Antibody (GAD or IA2) +ve	28 (27)	25 (28)	3 (20)	0.371

Data are mean±SD, n (%), or median (IQR) unless otherwise stated. P value is for Mann Whitney U test for continuous variables and Chi^2 test or Fisher's exact for the categorical variables. [a]Ethnicity was self-identified based on current official South African population group classification [b]History of other metabolic disease = history of hypertension, peripheral vascular disease, cerebrovascular disease, ischaemic heart disease, dyslipidaemia, polycystic ovarian syndrome or gestational diabetes. History of smoking and history of alcohol use was defined as any smoking or alcohol use at all in the year before admission.

ESM Table 2. Baseline characteristics of patients admitted with DKA, classified into Aβ groups

**Characteristic**	**A-β-**<br>(n = 22)	**A-β+**<br>(n = 41)	**A+β-**<br>(n = 16)	***A*+β+**<br>(n = 9)	***p*-value**
**Demographics**					
Age (years)	36±9	36±11	31±10	38 ±10	0.231
Male sex	16 (27)	19 (46)	9 (56)	6 (67)	0.216
Ethnicity					
Black	15 (68)	24 (59)	6 (38)	5 (56)	0.302
**Clinical history**					
Family history of diabetes	13 (59)	24 (59)	6 (38)	3 (33)	0.29
History of other metabolic disease[b]	5 (23)	8 (19)	2 (22)	1 (11)	0.8
HIV diagnosis	0	4 (10)	0	0	0.187
History of smoking	7 (32)	16 (39)	9 (56)	1 (11)	0.144
History of alcohol use	15 (68)	15 (37)	8 (50)	4 (44)	0.122
Precipitant identified	7 (32)	11 (27)	3 (19)	1 (11)	0.527
**Examination**					
BMI (kg/m^2)	26.0 (22.4-28.7)	32.7 (28.7-38.4)	23.1 (20.1-27.0)	34.0 (25.4-41.4)	<0.001
Acanthosis nigricans present	10 (46)	36 (88)	1 (6)	5 (56)	< 0.001
Systolic blood pressure (mmHg)	124±16	131±16	121±16	133±20	0.095
Diastolic blood pressure (mmHg)	77±10	83±12	73±11	80±8	0.045
**Admission Investigations**					
Glucose (mmol/l)	29.9 (25.8-44.0)	27.8 (23.8-36.5)	25.1 (20.2-40.5)	30.0 (24.6-47.5)	0.401
pH	7.14 (6.97-7.18)	7.19 (7.05-7.25)	7.06 (6.91-7.14)	7.15 (7.13-7.22)	0.016
Bicarb (mmol/l)	10.3 (5.7-12.4)	11.1 (7.4-13.9)	7.7 (3.9-9.0)	10.6 (8.4-11.9)	0.023

**Investigations**					
HbA1c (mmol/mol)	115±24	110± 21	110± 20	121± 15	
HbA1c %	12.72.2	12.2± 1.9	12.2± 1.8	13.2± 1.4	0.34
Creatinine (umol/l)	59.2± 17.8	64.1± 13.7	55.6± 13.7	71.2± 13.6	0.063
Total cholesterol (mmol/l)	4.9±1.0	4.8± 1.3	5.1± 0.8	4.8± 1.3	0.639
Triglycerides (mmol/l)	1.3±0.7	1.7± (0.7	1.1± 0.5	2.3± 0.9	0.002
HDL cholesterol (mmol/l	1.5± 0.6	1.1 ±0.	1.8± 0.8	1.1± 0.3	0.002
LDL cholesterol (mmol/l)	2.9± 0.9	2.9±1.2	2.8± 0.8	2.7± 1.2	0.934
**Outcome**					
Off insulin at 12 months	9 (41)	28 (68)	0	3 (33)	< 0.001
Off insulin at 60 months	6 (27)	15 (37)	0	2 (22)	0.04

Data are mean±SD, n (%), or median (IQR) unless otherwise stated. P value is for Mann Whitney U test for continuous variables and Chi^2 test or Fisher's exact for the categorical variables. [a]Ethnicity was self-identified based on current official South African population group classification [b]History of other metabolic disease = history of hypertension, peripheral vascular disease, cerebrovascular disease, ischaemic heart disease, dyslipidaemia, polycystic ovarian syndrome or gestational diabetes. History of smoking and history of alcohol use was defined as any smoking or alcohol use at all in the year before admission.

A+ = IA2 or anti-Gad positive; β+ = beta cell positive (fasting c-peptide > 0.3 nmol/l)

ESM Figure 1. HbA_{1c} over time between AB subgroups

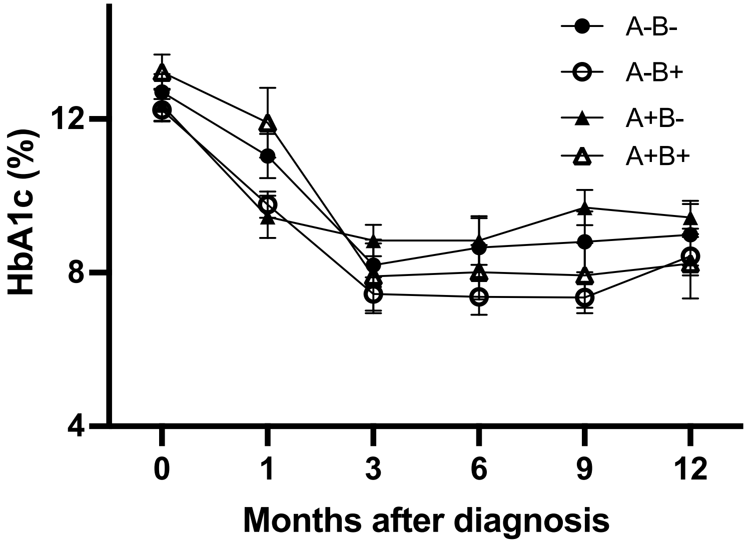

A+ = IA2 or anti-GAD positive; B+ = beta cell positive (fasting c-peptide > 0.3nmol/l)
